# How to control single-molecule rotation

**DOI:** 10.1038/s41467-019-12605-8

**Published:** 2019-10-11

**Authors:** Grant J. Simpson, Víctor García-López, A. Daniel Boese, James M. Tour, Leonhard Grill

**Affiliations:** 1 Department of Physical Chemistry, University of Graz, Heinrichstrasse 28, 8010 Graz, Austria; 2 Departments of Chemistry and Materials Science and NanoEngineering, and the Smalley-Curl Institute and NanoCarbon Center, Rice University, Houston, TX 77005 USA; 3 Department of Theoretical Chemistry, University of Graz, Heinrichstrasse 28, 8010 Graz, Austria

**Keywords:** Scanning probe microscopy, Molecular machines and motors, Imaging techniques, Surfaces, interfaces and thin films

## Abstract

The orientation of molecules is crucial in many chemical processes. Here, we report how single dipolar molecules can be oriented with maximum precision using the electric field of a scanning tunneling microscope. Rotation is found to occur around a fixed pivot point that is caused by the specific interaction of an oxygen atom in the molecule with the Ag(111) surface. Both directions of rotation are realized at will with 100% directionality. Consequently, the internal dipole moment of an individual molecule can be spatially mapped via its behavior in an applied electric field. The importance of the oxygen-surface interaction is demonstrated by the addition of a silver atom between a single molecule and the surface and the consequent loss of the pivot point.

## Abstract (web summary)

The orientation of a molecule on a surface affects many processes, so the ability to control single-molecule rotation could be powerful. Here, the authors use the electric field from a scanning tunneling microscope tip to precisely induce unidirectional rotation of a polar molecule, allowing visualization of the molecule’s internal dipole moment.

## Introduction

Controlling the motion and orientation of single molecules is key to the understanding of heterogeneous catalysis, growth and assembly processes as well as to the operation of surface-adsorbed molecular machines. Specifically, the orientation of a molecule or a functional group can affect many processes, for instance, molecular diffusion on a surface^1,2^, the efficiency of a chemical reaction^3,4^, and heterogeneous catalysis^5^ or biochemical reactions with enzymes as catalyst^6^. Deterministically controlling the direction of molecular rotation and translation remains a challenge because numerous degrees of freedom exist and thermally activated processes offer little selectivity^7^. Scanning tunneling microscopy (STM) is attractive in this regard as it provides the means to image and manipulate matter at the single-molecule scale and track thermally induced processes like rotation and translation on metallic surfaces^8^. Moreover, the STM tip has been used to induce rotation of various molecules^9–11^. While rotation is often combined with translation, e.g., along the edge of a molecular island^12^, it can also be constrained to a fixed rotational axis^13,14^. Molecular rotors in double-decker complexes have been rotated in large assemblies, albeit without control over the direction of rotation^15^.

**Fig. 1:**
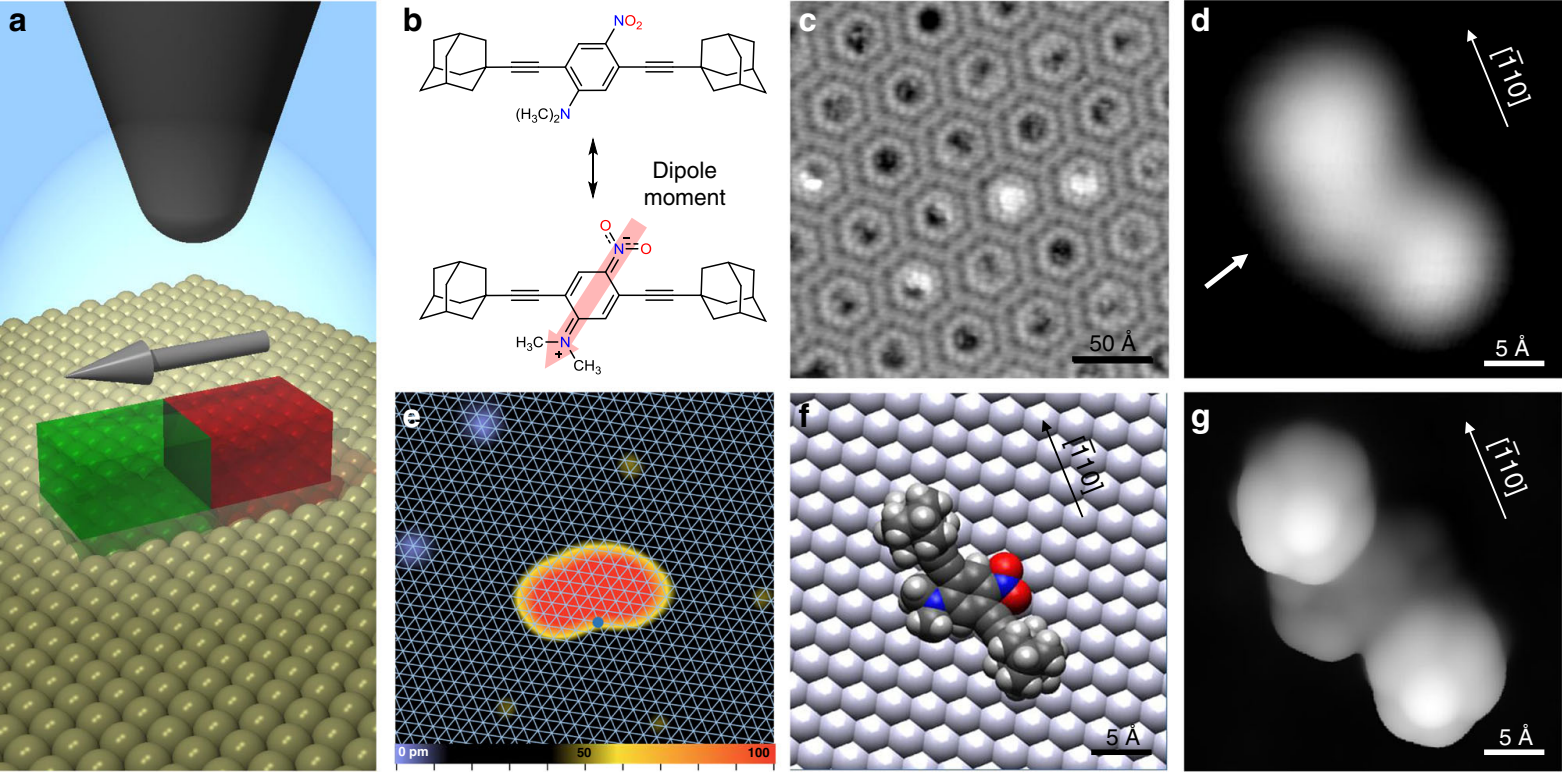
Adsorption of polar DDNB molecules on Ag(111). **a** Scheme of the experimental setup. **b** Chemical structures of DDNB showing the permanent net dipole moment from the negatively charged –NO_2_ to the positively charged -N(CH_3_)_2_ groups. **c** STM image (1.03 V, 0.76 pA) of highly ordered DDNB assemblies on Ag(111) (see Supplementary Fig. 1). **d** STM image (1.03 V, 0.27 pA) of a single DDNB molecule and **e** (0.70 V, 0.27 pA) of a larger area with Ag adatoms (yellow) and CO molecules (blue) with respect to the underlying Ag(111) lattice. The pivot point of the molecule is marked by a blue spot. The color scale indicates apparent height. **f** Calculated adsorption geometry on the Ag(111) surface. **g** Simulated STM image based on the optimized structure shown in **f**

In contrast to non-deterministic processes, we show that the electric field in an STM junction can be used to manipulate polar molecules with absolute precision (Fig. 1a). Here, we manipulate single dipolar molecules and obtain unidirectional rotation on demand, and thus deterministic behavior. From these precision manipulation experiments, the electric field-induced motion can be mapped and used to determine the internal dipole moment of a single molecule.

## Results

### Molecular adsorption on Ag(111)

We have developed the 2,5-di(ethynyladamantanyl)−4-(dimethylamino)nitrobenzene molecule (DDNB)^16^ which, at its core, has a strong electric dipole. This arises from the electron-donating character of the dimethylamine (−N(CH_3_)_2_) and the electron-withdrawing properties of the nitro (−NO_2_) group attached to the central benzene ring (Fig. 1b) with a resulting net dipole moment of 6.78 Debye in the gas phase (see Supplementary Table 1). To protect the polar groups from strong adsorption with the surface, DDNB contains two peripheral adamantane groups which allows for facile diffusion and interaction with the electric field^16^. Depositing these molecules onto a Ag(111) surface at room temperature, results in highly ordered honeycomb assemblies (Fig. 1c) with ordering determined by the dipolar interaction between molecules (Supplementary Fig. 1).

Deconstructing the islands molecule by molecule (Supplementary Fig. 2) allows individual isolated molecules to be investigated. They appear with two lobes at a distance of 13.0 ± 0.1 Å (Fig. 1d), which we assign to the adamantane groups. The peanut-shape asymmetry of the molecule comes from the polar −N(CH_3_)_2_ and −NO_2_ substituents of the central benzene ring. Taking into account the dimethylamine-nitro axis with respect to the adamantane axis, the surface imposes a chirality on the molecule^17^ and two enantiomers exist on the surface, identified via a slight protrusion on the convex side of the molecule (arrow in Fig. 1d). Only one of the two enantiomers is considered in the following (see Supplementary Fig. 3).

The adsorption site of the molecule was determined by placing single silver adatoms in the vicinity of a molecule^18^. Additionally, CO molecules are identified as depressions (blue in Fig. 1e) from their characteristic size (diameter of ~0.8 nm)^19^. As adatoms adsorb in hollow^20^ and CO in on-top sites^21^ on Ag(111), we can superimpose the silver lattice onto an STM image, thus deriving the position of a single DDNB molecule with respect to the surface (Fig. 1e). Further, the computationally determined geometry (Fig. 1f) reveals that the axis between the two adamantane groups lies ~18° rotated with respect to the $[\bar 110]$ direction of the surface. From this calculated structure, an STM image was simulated (Fig. 1g) that correlates well to the experimental result. One can identify that the adamantane groups reflect the two large lobes of the STM image while the dimethylamine is responsible for the asymmetric appearance, indicated by the arrow in Fig. 1d.

### Controlled rotation around a fixed pivot point

**Fig. 2:**
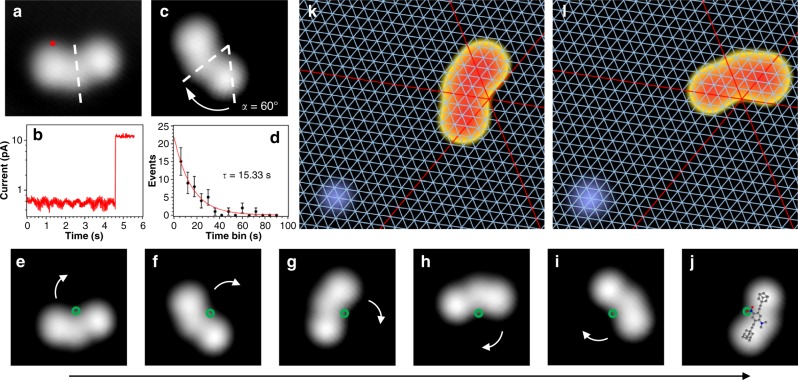
Controlled rotation of single molecules. STM images (1.03 V, 0.27 pA) before (**a**) and after (**c**) a voltage pulse ( + 1.31 V at the position marked in red). **b** Current recorded as a function of time with open feedback loop during the pulse in **a**. The step corresponds to a rotation of the molecule. **d** Poisson distribution of times t before a rotation occurred, fitted with $A \cdot e^{ - t/\tau }$ (error bars represent the standard deviation). **e**–**j** Series of consecutive STM images (1.03 V, 0.27 pA) with a complete clockwise rotation (counter-clockwise rotation in Supplementary Fig. 9) by voltage pulses with the tip always in the very same position with respect to the molecule (indicated in **a** by a red dot), thus in six positions with respect to the surface. **k**–**l** STM images (0.70 V, 0.27 pA) of a molecule before and after rotation (α = 60°) with CO molecule (blue, lower left) as reference point. The point of intersection of the red lines indicates a top site of the Ag(111) surface as pivot point (marked green in **e**–**j**)

Very low tunneling currents (<1 pA) are required for stable imaging as the molecules otherwise move during scanning. To induce this motion in a controlled fashion we applied a voltage pulse of 1.3 V at the location indicated in Fig. 2a. A sudden increase in the tunneling current (Fig. 2b) and subsequent imaging (Fig. 2c) reveals that the molecule rotated clockwise by the azimuthal angle *α* = 60° (Fig. 2c). Hence, the molecule ends in an orientation equivalent to before the rotation (with respect to the top-most surface layer). Each rotational event can be considered an independent process since they can be described by a Poisson distribution (Fig. 2d) with an extracted rate (1/τ) of 0.07 s^−1^ if the tip is placed as indicated in Fig. 2a. Note that already at voltages of about 1.5 V and above, molecular translation occurs in addition to the rotation, rendering the window of suitable bias voltages rather narrow and impeding a systematic variation of the bias voltage.

If several such identical manipulations are performed in a sequence, an individual molecule can be rotated unidirectionally in a highly controlled manner. Specifically, six orientations are found during one entire cycle of clockwise rotation (Fig. 2e–j), in agreement with the six-fold rotational symmetry of the surface layer (note that rotational angles different from multiples of 60° were never observed). Indeed, nudged elastic band calculations confirm this 60° periodicity since a single barrier without further minima is found between two stable orientations (see Supplementary Fig. 4). Importantly, by comparing individual images before and after a rotational event, we find that there is a single common pivot point (green circle in Fig. 2e–j) located on the −NO_2_ group. By superimposing the chemical structure over the final STM image (Fig. 2j), it becomes clear that the pivot point of molecular rotation corresponds to one of the two oxygen atoms of the −NO_2_. Hence, this specific oxygen-silver bond acts as an anchor during the rotation, similar to the rotation of butyl methyl sulfide molecules around a sulfur-metal bond on Cu(111)^14^.

When imaging in the presence of a reference point on the surface (CO molecule), the three close-packed atomic rows of the surface (red lines in Fig. 2k–l) cross exactly in the position of the pivot point. Therefore, we can unambiguously identify a single oxygen atom positioned in an on-top site of the Ag(111) surface as the pivot point (see Fig. 1e, details in Supplementary Fig. 5). Furthermore, when calculating the distances of each atom in the molecule from the surface (Supplementary Fig. 4), it is revealed that the smallest distance, i.e., strongest molecule-surface interaction, is from the central molecular oxygen atom to a Ag(111) top site, in agreement with experiments. Note that the pivot point is determined by a specific chemical interaction between the molecule and an atomically flat fcc(111) surface. This is in contrast to reconstructed surfaces such as Au(111) where the elbows of the herringbone reconstruction can act as a “physical” pivot point since they are preferred adsorption sites for any molecule.

**Fig. 3:**
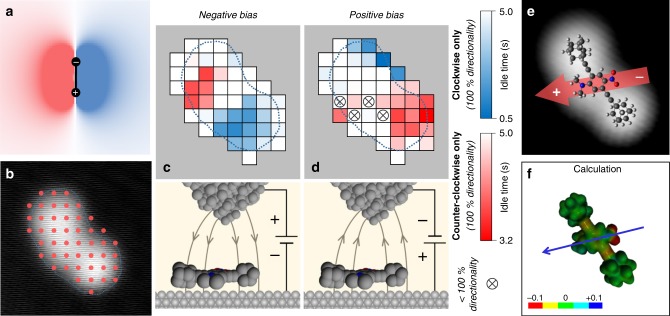
Mapping the dipole moment in a single molecule. **a** Simulation of the torque acting on an electric dipole (shown in black) from a positive test charge placed at different locations (see Supplementary Fig. 6 for details). Blue (red) areas represent clockwise (counter-clockwise) rotation of the dipole, respectively and the contrast indicates the magnitude of torque. **b** Voltage pulses were applied in 46 positions (red dots) over a single molecule. If the resulting rotation is clockwise or counter-clockwise, the pixel is colored blue or red, respectively. **c**, **d** Rotational maps for negative and positive sample bias with legend to the right (crossed circles if both directions of rotation occurred) and schematic representations of the electric field below. **e** Orientation of the internal dipole deduced from the maps in **c**, **d**, which is in agreement with the calculated charge distribution and dipole (blue arrow) of the adsorbed molecule (**f** colors give different potentials in units of elementary charge **e**, see Supplementary Fig. 8)

Before we discuss the molecular rotation in detail, it is useful to consider the forces on an electric dipole, treated as a rigid object composed of two point charges, in the presence of a (positive) point charge. We have used a simple simulation to obtain the qualitative behavior of an electric dipole in the two-dimensional confinement of the surface, but did not consider screening effects of the surface that might cause quantitative deviations. The model (for details see Supplementary Fig. 6), which includes varying positions of the point charge in a two-dimensional plane, results in two well-defined regimes of torque on the dipole, clockwise (indicated in blue) and counter-clockwise rotation (red), separated along the direction of the dipole moment (Fig. 3a). Hence, in an STM setup where a local (inhomogeneous) electric field is applied in the vicinity of the molecule, a similar trend can be expected depending on tip position and polarity.

### Visualization of the molecular dipole

When changing the lateral tip position with respect to the molecule in the experiment, the rate of rotation varies between low values of ~0.02 s^−1^ up to 16.6 s^−1^ (Supplementary Fig. 7), and decays quickly if the tip is not above the molecule. Spatial rate dependence is expected for many mechanisms in STM^22,23^ but crucially, also the direction of DDNB rotation (counter-clockwise/clockwise) depends on the tip location. To reveal the underlying process, we systematically varied the tip position with respect to the molecule (Fig. 3b). By plotting average idle time at each pixel in different colors as well as different intensities, we can map both direction and efficiency of a rotation.

The result using negative sample bias (Fig. 3c) shows that the direction of rotation clearly depends on which side of the dimethylamine-nitro axis the STM tip is placed. That is, with the STM tip over the lower right half of the molecule, solely clockwise rotation is induced (blue area) and counter-clockwise rotation (red area) occurs on the other side. This agrees with the expectations from an idealized setup (Fig. 3a) for a positive test charge, in analogy to the STM setup where the chosen bias voltage polarity results in an electric field pointing away from the tip (bottom of Fig. 3c). Apparently, only two dimensions are sufficient in the simulation since molecular motion is constrained to the surface plane, and therefore the electric field components parallel to the surface dominate.

Importantly, for the same tip position the rotation direction is inverted when positive bias voltage is applied. Now, the right side of the molecule is red while the other side is mainly blue (Fig. 3d). We can exclude differences in the STM tip to be responsible because the entire series in Fig. 3b–d was done with the same molecule and no tip modifications. Additionally, the same qualitative behavior has been reproduced for all molecules studied or after a tip change has occurred, for instance by controlled indentation into the surface to improve its quality.

While the qualitative agreement between experiment and theory is very good, the unequal distribution of rotational rates over the molecule when comparing the two polarities (Fig. 3c–d) deviates from the simulation’s highly symmetric butterfly shape. Plausible reasons are the varying interaction strength with the surface along the dipole moment (being strongest at the pivot point), the spatial charge distribution within the molecule (in contrast to point charges), and the inhomogeneity of the electric field in the STM junction.

Molecular motion can be induced by STM manipulation in various ways: resonant tunneling^22^, inelastic electron tunneling^9^, chemical forces^24^, or electric fields^25^. For molecules adsorbed on an insulating film, resonant tunneling can cause temporary charging, which then leads to molecular translation driven by the electrostatic field in the STM junction^26^. Moreover, while a molecular motion may be caused by inelastic tunneling, an electric field may – in addition – control the direction of motion as has been observed for single-molecule translation^27,28^. Based on the rotational behavior in DDNB, only the effect of the electric field can explain the observed directionality. Specifically, the rotation direction of the molecules is reversed (Fig. 3c–d) if *(i)* the tip crosses the molecular axis (with the same bias polarity) or *(ii)* the bias polarity is changed (at fixed tip position). In both cases the lateral component of the electric field is inverted at the position of the dipole moment, and consequently the resulting torque is also inverted. While it is possible that inelastic tunneling may play a role in inducing the rotation, we strongly believe that the rotation is dominated by the electric field in the STM junction, because the distribution of rotational rates over the molecule is unequal for the two polarities (Fig. 3c–d). Note that very similar distributions are expected for inelastic tunneling where efficiencies depend on the tip position over the molecule^29^ and this spatial dependence should be comparable for both bias polarities^30^.

The origin for this electric field-induced rotation is the permanent molecular dipole moment. If it were dominated by an induced dipole moment, the same direction of rotation would be observed, regardless of the bias voltage polarity used (at the same location over the molecule). Hence, rotation of an electric dipole (around a fixed axis) in an external field is induced at low bias while translation occurs in addition at high bias. This is an advancement to the electric monopoles of negatively charged CH_3_S molecules, which hop away from a negatively charged STM tip and towards a positively charged one, respectively^27^, because the electric field can cause only translation. Consequently, from this molecular response to the applied electric field, the location and direction of the internal dipole moment can be assigned (Fig. 3e), which turns out to be in perfect agreement with the calculated charge distribution over the molecule (Fig. 3f).

Due to the very high level of control over the individual rotations in the present case, any orientation and any sequence of rotational steps, clockwise (Fig. 2e–j) or counter-clockwise (Supplementary Fig. 9), can be achieved at will. Hence, control is maximized to 100% directionality. This is defined as |*N*_clockwise_ − *N*_counter-clockwise_|/*N*_total_; i.e., 0% in the complete random case and 100% for exclusively one direction of rotation. This is much higher than previously reported values of between 5%^14^ and ~56%^10^. Note that the STM tip position was fixed in these experiments and the situation is very different if lateral tip motion is allowed because the molecular position within the local potential landscape is continuously modified^31^.

### Disruption of the pivot point

**Fig. 4:**
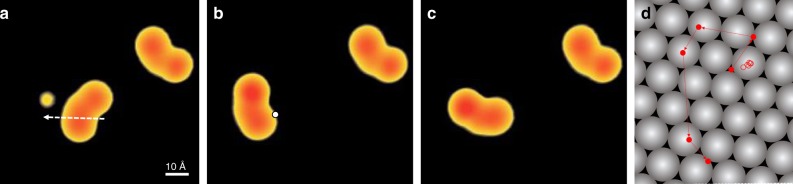
Pivot point destabilization with a single adatom. **a** STM image (0.73 V, 0.32 pA) of two DDNB molecules and a single Ag adatom on Ag(111). The dotted arrow indicates the pathway of a lateral manipulation to move the lower molecule onto the adatom. **b**–**c** Subsequent STM images of the same surface area. A voltage pulse from the STM tip (at the white dot) results in molecular motion, which is no longer centered on the pivot point. The same contrast scale is used in **a**–**c**. **d** The difference in molecular motion during STM manipulation is evident from the location of the molecule during sequences of successive pulses (red circles indicate the position of the NO_2_ group as defined in Supplementary Fig. 5; the Ag(111) surface is shown in gray). While a fixed axis of rotation is found on the flat surface (open circles), as presented in Fig. 2e–j, a single adatom underneath the molecule destabilizes this axis and results in translation in addition to rotation (solid circles). The complete image series is shown in Supplementary Fig. 10

The high rotational control around a well-defined pivot point is only present because of the specific interaction between the NO_2_ group and the surface. If this interaction is disrupted, the fixed axis of rotation should be lost. Actually, our experiments show that the addition of only one extra adatom between the molecule and surface is sufficient to do so. When moving a single DDNB molecule on a silver adatom by lateral manipulation (Fig. 4a), the molecular appearance changes only little with one of two lobes becoming slightly brighter (Fig. 4b). Upon inducing rotation (in the same way as in Fig. 2), we find that the molecule does not only rotate but also translates (Fig. 4c). This is more evident when following the very same molecule during a sequence of manipulation events (Fig. 4d), either with or without a silver atom underneath. In the latter case the pivot point remains close to the same on-top site during the entire sequence (open circles). However, in the former case, it is accompanied by substantial translations of the molecule (solid circles). Hence, the fixed pivot point is lost due to the presence of the silver atom.

Here we have shown complete control of the rotation direction, and therefore the orientation, of polar molecules and this leads us to a deeper understanding of how molecules navigate potential energy landscapes. Further, mapping the rotation behavior over the spatial extent of a single-molecule has allowed a direct visualization of the internal molecular dipole. By modifying the specific molecule-surface interaction with single adatoms, the rotational axis can be destabilized and translation occurs. Understanding such processes is also of interest in view of chemical reactions that are accelerated by local electric fields^32^ or internal molecular motors^33^ that involve photo-excited states and thus non-continuous charge distributions within the molecule. Using this method, a programmed approach might be developed and further refined by machine learning, to move molecules in large numbers for controlled goals.

## Methods

### Experimental

The Ag(111) surface was cleaned by repeated cycles of sputtering with Ar^+^ followed by annealing to ~770 K. Deposition of DDNB was done via sublimation from a crucible held at 389 K into the UHV chamber (base pressure ~1 × 10^−10^ mbar). The prepared sample was then cooled to 7 K before STM measurements. All STM images were acquired using a commercial CreaTec instrument in the constant-current mode. The STM tip is an electrochemically etched tungsten wire. To ensure clear high-resolution imaging the tip is subsequently sharpened through high-voltage (100 V) tip forming which leaves a Ag tip termination.

## Supplementary information

Supplementary Information

## Data Availability

Data from this study are available from the corresponding authors upon request.

## References

[1] Otero R (2004). Lock-and-key effect in the surface diffusion of large organic molecules probed by STM. Nat. Mater..

[2] Rotter P (2016). Coupling between diffusion and orientation of pentacene molecules on an organic surface. Nat. Mater..

[3] Chang Y-P (2013). Specific chemical reactivities of spatially separated 3-aminophenol conformers with cold Ca+ ions. Science.

[4] Brandt M, Greber T, Böwering N, Heinzmann U (1998). The role of molecular state and orientation in harpooning reactions: N2O on Cs/Pt(111). Phys. Rev. Lett..

[5] Brandt K, Chiu ME, Watson DJ, Tikhov MS, Lambert RM (2009). Chemoselective catalytic hydrogenation of acrolein on Ag(111): Effect of molecular orientation on reaction selectivity. J. Am. Chem. Soc..

[6] Koshland DE (1956). Molecular geometry in enzyme action. J. Cell. Comp. Physiol..

[7] Barth JV (2000). Transport of adsorbates at metal surfaces: from thermal migration to hot precursors. Surf. Sci. Rep..

[8] Wong KL, Kwon K-Y, Bartels L (2006). Surface dynamics of benzenethiol molecules on Cu(111). Appl. Phys. Lett..

[9] Stipe BC, Rezaei MA, Ho W (1998). Inducing and viewing the rotational motion of a single molecule. Science.

[10] Perera UGE (2012). Controlled clockwise and anticlockwise rotation switching of a molecular motor. Nat. Nanotech.

[11] Lensen S, Elemans JAAW (2012). Artificial molecular rotors and motors on surfaces: STM reveals and triggers. Soft Matter.

[12] Chiaravalloti F (2007). A rack-and-pinion device at the molecular scale. Nat. Mater..

[13] Gimzewski JK (1998). Rotation of a Single Molecule Within a Supramolecular Bearing. Science.

[14] Tierney HL (2011). Experimental demonstration of a single-molecule electric motor. Nat. Nanotechnol..

[15] Zhang Y (2016). Simultaneous and coordinated rotational switching of all molecular rotors in a network. Nat. Nanotechnol..

[16] Simpson GJ, Garcia-Lopez V, Petermeier P, Grill L, Tour JM (2017). How to build and race a fast nanocar. Nat. Nanotechnol..

[17] Mark AG, Forster M, Raval R (2010). Direct visualization of chirality in two dimensions. Tetrahedron-Asymmetry.

[18] Lagoute J, Kanisawa K, Fölsch S (2004). Manipulation and adsorption-site mapping of single pentacene molecules on Cu(111). Phys. Rev. B.

[19] Kulawik M (2005). Interaction of CO molecules with surface state electrons on Ag(111). Surf. Sci..

[20] Ratsch C, Seitsonen AP, Scheffler M (1997). Strain dependence of surface diffusion: Ag on Ag(111) and Pt(111). Phys. Rev. B.

[21] Gajdos M, Eichler A, Hafner J (2004). CO adsorption on close-packed transition and noble metal surfaces: trends from ab initio calculations. J. Phys.: Condens. Matter.

[22] Lastapis M (2005). Picometer-scale electronic control of molecular dynamics inside a single molecule. Science.

[23] Grill L (2006). Exploring the interatomic forces between tip and single molecules during STM manipulation. Nano Lett..

[24] Keeling DL, Humphry MJ, Moriarty P, Beton PH (2002). Attractive mode manipulation of covalently bound molecules. Chem. Phys. Lett..

[25] Rezaei MA, Stipe BC, Ho W (1999). Atomically resolved adsorption and scanning tunneling microscope induced desorption on a semiconductor: NO on Si(111)-(7x7). J. Chem. Phys..

[26] Swart I, Sonnleitner T, Niederführ J, Repp J (2012). Controlled lateral manipulation of molecules on insulating films by STM. Nano Lett..

[27] Ohara M, Kim Y, Kawai M (2008). Electric field response of a vibrationally excited molecule in an STM junction. Phys. Rev. B.

[28] Pawlak R (2017). Design and characterization of an electrically powered single molecule on gold. ACS Nano.

[29] Stipe BC, Rezaei MA, Ho W (1999). Localization of inelastic tunneling and the determination of atomic-scale structure with chemical specificity. Phys. Rev. Lett..

[30] Torres JAG, Simpson GJ, Adams CJ, Früchtl HA, Schaub R (2018). On-demand final state control of a surface-bound bistable single molecule switch. Nano Lett..

[31] Manzano C (2009). Step-by-step rotation of a molecule-gear mounted on an atomic-scale axis. Nat. Mater..

[32] Aragonès AC (2016). Electrostatic catalysis of a Diels-Alder reaction. Nature.

[33] Klok M (2008). MHz unidirectional rotation of molecular rotary motors. J. Am. Chem. Soc..

