# Supplementary material for: How to control single-molecule rotation
Source: Nat Commun. 2019 Oct 11;10:4631. doi: 10.1038/s41467-019-12605-8 (PMC6788993; doi:10.1038/s41467-019-12605-8)
Supplement: Supplementary file 1 — Supplementary Information [file 41467_2019_12605_MOESM1_ESM.pdf]

How to Control Single-Molecule Rotation

Grant J. Simpson[1], Víctor García-López[2], A. Daniel Boese[3], James M. Tour[2]*, and Leonhard Grill[1]*

1) Department of Physical Chemistry, University of Graz, Heinrichstrasse 28, 8010 Graz, Austria.

2) Departments of Chemistry and Materials Science and NanoEngineering, and the Smalley-Curl Institute and NanoCarbon Center, Rice University, Houston, Texas 77005, United States.

3) Department of Theoretical Chemistry, University of Graz, Heinrichstrasse 28, 8010 Graz, Austria.

**Correspondence to: J.M.T. (tour@rice.edu) and L.G. (leonhard.grill@uni-graz.at)*

Contents

Supplementary Methods S2
- Molecular islands S2
- Surface chirality S4
- DFT methods S5
- Pivot point determination S7
- Dipole simulation S7
- Inducing rotation by voltage pulses S8
- Electrostatic potential S9
- Complete rotation sequences in both directions S9
- Rotation sequences with and without Ag Adatom S10
- Synthesis of 2,5-di(ethynyladamantanyl)-4-(dimethylamino)nitrobenzene S11

Supplementary References S16

Supplementary Methods

Molecular islands

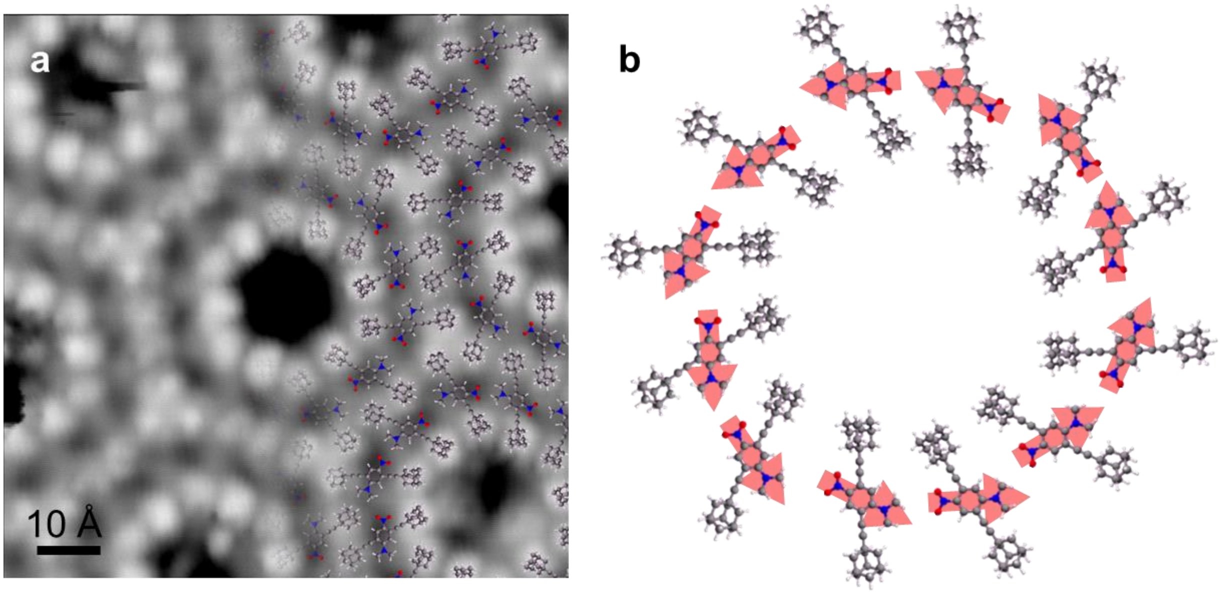

Supplementary Figure 1: Intermolecular interactions. *(a) Constant current STM image of single hexagonal unit with overlaid chemical structure indicating the position and orientation of individual molecules (I = 0.8 pA, V = 1.64 V). (b) The 12 constituent molecules of a single pore with red arrows indicating the dipole moments.*

Upon deposition of DDNB onto a Ag(111) surface at room temperature, the molecules form highly-ordered hexagonal porous structures (Fig. 1c). Pores are separated by a distance of 47.3 Å and are observed both empty and occupied by further single molecules. It is not immediately obvious how the individual molecules are arranged to form these islands.

From the controlled deconstruction of a molecular assembly (see Supplementary Figure 2), we can assign one lobe of a single molecule to a feature on the inner hexagon of a single unit cell and the other lobe to the outer hexagon as shown in Supplementary Figure 1a. The unit cell consists of 12 molecules which interdigitate with the molecules of the directly neighbouring cells. A closer look at the region between the inner and outer hexagons of a single unit cell reveal alternating bright and dark features. Using the lobes as a guide on how individual molecules are oriented one can see that the bright features match well with the position of the dimethylamine groups and the dark features correspond to the position of the nitro groups of individual molecules. This assignment is also in agreement with the case of the isolated molecule whereby we see a protrusion on the convex side of the molecule corresponding to the dimethylamine, and opposite, a concave hollow feature corresponding to the nitro group.

In this configuration, internal dipoles of the molecules are thus arranged end-to-end as shown in Supplementary Figure 1b. We can therefore understand the close-packed arrangement as a result of favourable dipole-dipole interactions between the polar groups of individual molecules. In addition, the non-polar admantane groups are arranged so that the maximum van der Waals interaction exists (i.e. all adamantine cages pointing inwards towards pore, or, outwards towards other adamantine groups of neighbouring unit cells).

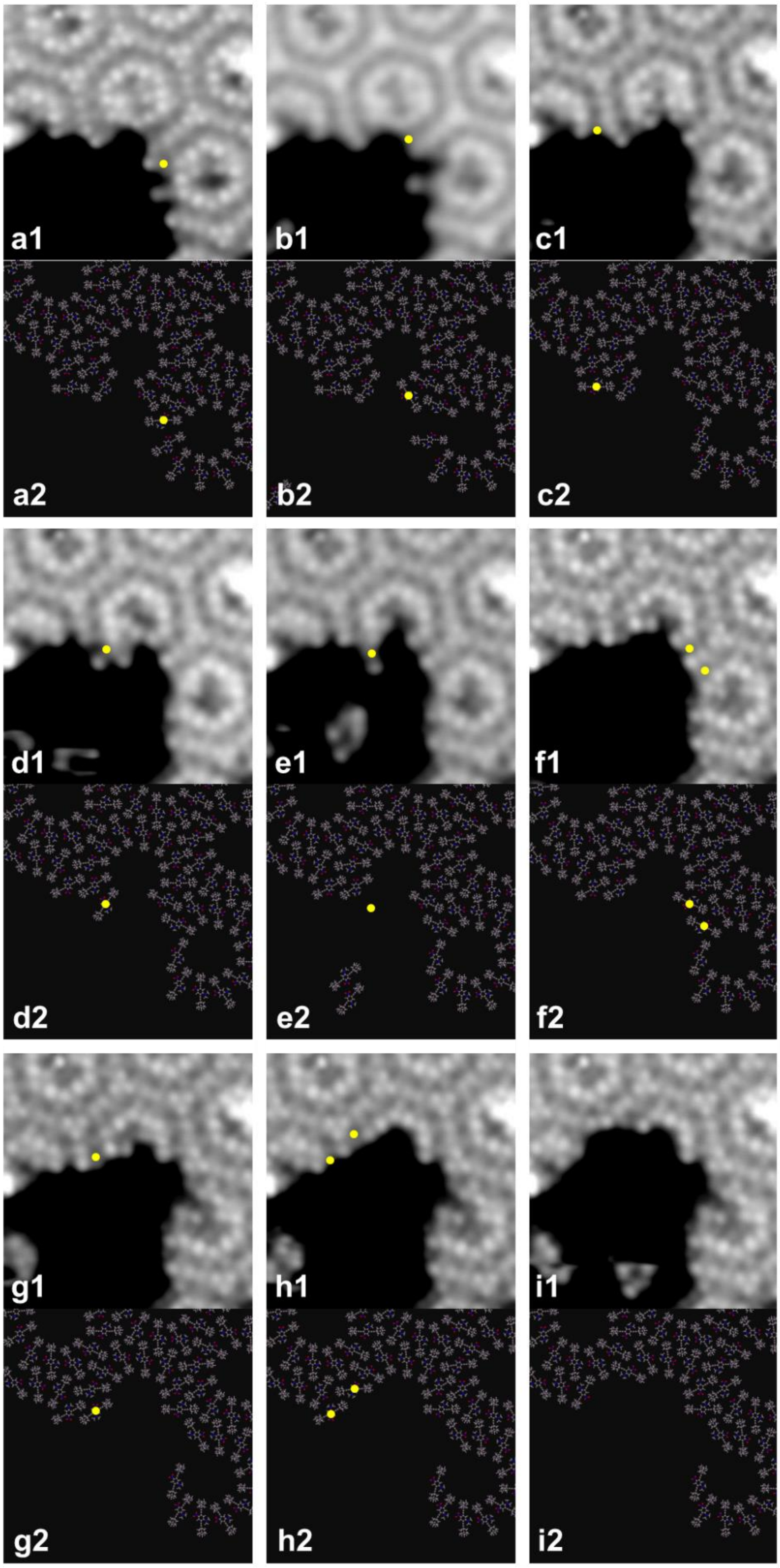

Supplementary Figure 2: Deconstruction of self-assembled molecular islands. *Series of STM images (1, a-i) (I = 0.4 pA; V = 1.04 V; 11 nm x 11 nm) and corresponding models of the molecular assemblies (2, a-i). The yellow dots indicate the molecules that have been laterally manipulated between each consecutive image.*

In order to determine the structure, a series of lateral STM manipulations was performed to deconstruct an island molecule-by-molecule (Supplementary Figure 2). Relatively gentle manipulation parameters (constant current; 0.1 nA; 0.1 V) were used to allow controlled removal of molecules from the islands. In all cases, the lateral manipulation starts at the location of the marked molecule(s) and is directed towards the lower left region of the image. Typically, single molecules (marked in yellow in Supplementary Figure. 2) are removed from the islands and can be seen to consist of two lobed structures corresponding to the two admantane groups. In some cases one can see that the manipulated molecule has likely been transferred to the tip (for example in Supplementary Figure 2a-b the molecule indicated has been removed from the island but the appearance of the remaining island has changed). In other cases, the molecule is simply removed from the island and can be seen in the lower left region where the manipulation ended (e.g. Supplementary Figure 2d-e).

Surface chirality

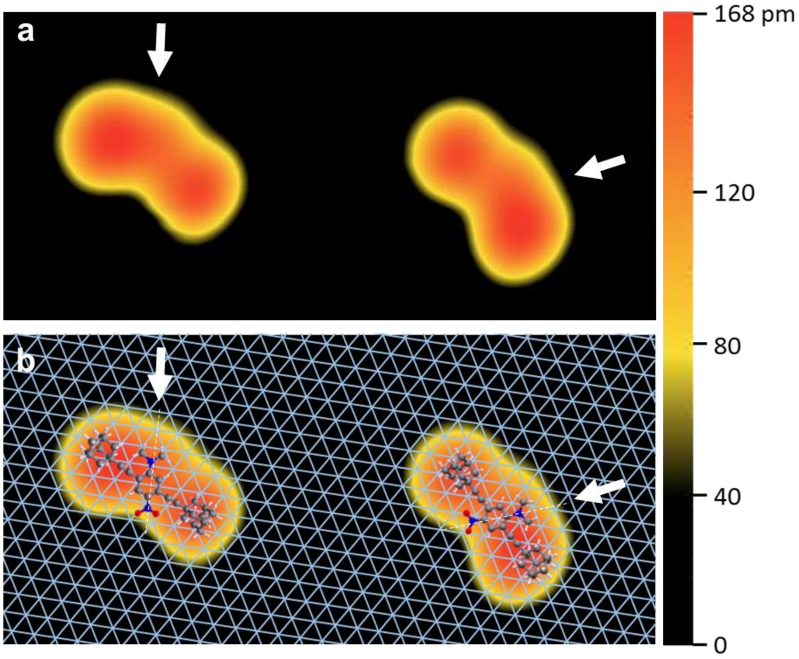

Supplementary Figure 3: Surface chirality. *(a) Constant current STM image of two neighbouring molecules of differing chirality. The arrows indicate the subtle difference in apparent height to the left or right side of the molecular axis. The overlaid chemical structure in (b) illustrates that this is due to the orientation of the nitro – dimethylamine axis in each of the surface enantiomers. (STM image: 86 Å × 44 Å; I = 0.27 pA; V = -0-70 V). The colour scale is given at the right with the corresponding apparent heights.*

Although no chiral centre exists within the (gas phase) molecule, the reduction in symmetry upon adsorption on a surface results in two distinct (but degenerate) enantiomers. In STM images these are distinguished by a slight asymmetry on the convex edge of the molecule (arrows in Supplementary Figure 3a). This asymmetry corresponds well to the orientation of the axis along which the nitro and dimethylamine groups are aligned (Supplementary Figure 3b). With both enantiomers, six rotational orientations, each separated by 60°, are observed.

Note that only one of the two enantiomers, i.e. the one at the right in Supplementary Figure 3 (and Fig. 1d), is considered in our study in order to allow direct comparison of different manipulation experiments. It should be mentioned that in our experiments both enantiomers rotated in the same fashion (described in Fig. 2).

DFT methods

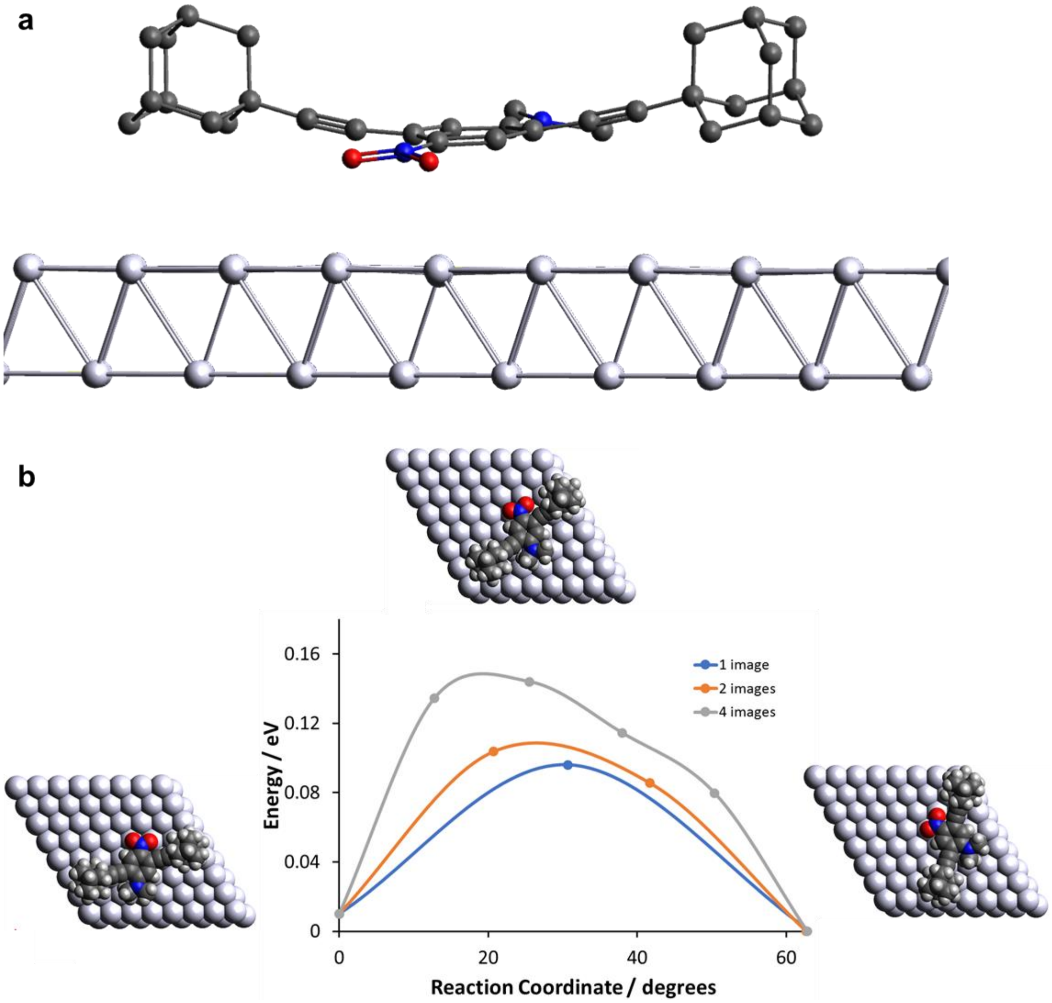

Supplementary Figure 4: Surface-Interaction and Rotation of the Molecule. *(a) Distances of the heavy atoms of DDNB towards the nearest-neighbour surface atoms, correlating with the interactive strength of the respective functional groups. (b) Climbing-image nudged elastic band showing reaction pathway from initial (left) to final (right) low-energy configurations via a transition state (top) of energy ~0.1 eV.*

For the computation of the dipole moments of DDNB (Supplementary Table 1), the TURBOMOLE suite of programs has been employed[5] using a large gaussian basis set of triple-zeta quality.[6] Both PBE and B3LYP[7,8] functionals basically yield the same dipole moments, indicating that there is little change by the DFT methodology used.

		B3LYP/TZVPPD	PBE/TZVPPD
**gas phase**		6.71	6.78
**on the surface**	total dipole moment	8.36	8.48
	component parallel to the surface	7.96	8.00
	component perpendicular to the surface	2.55	2.80

Supplementary Table 1: Dipole Moments. *Dipole moment in Debye of DDNB either optimised in the gas phase or in its surface geometry.*

For the DFT calculations of the surface and the simulated STM images, we employed the VASP program.[1] Plane-wave basis sets with an energy cut-off of 500 eV have been utilized together with the standard PAW potentials of the VASP program[2] for N, O, C, and H, and for Ag, the semicore p states have been treated as valence. The energy has been converged to 10^{-7} eV and the gradients to 0.005 eV/Ångstrom.

**2 layers**		**3 layers**	**best estimate**
8×8	10×10	8×8	
283.4 kJ mol^{-1}	255.5 kJ mol^{-1}	285.9 kJ mol^{-1}	258.0 kJ mol^{-1}

Supplementary Table 2: Adsorption Energies. *Adsorption energy of DDNB on Ag(111). 8×8 and 10×10 represent the lateral size of the surface area, i.e. silver atoms on the surface slab.*

We furthermore computed several orientations of the molecule on the surface to ensure to have computed the minimum geometry. For all surface calculations, the PBE functional[3] has been used together with the D3 dispersion.[4] The distance in z-direction was chosen to be 25 Ångstrom, leaving sufficiently enough space between the slabs. For the z-direction of the slab, the dipole moment has been corrected for. The silver-silver distance was chosen to be 2.83275 Ångstrom, each time leaving the lowest layer of the Ag(111) surface fixed and the relaxing the upper layer(s). All calculations have been performed at the Γ point.

The adsorption energy of several slab sizes using either 2 or 3 layers of silver are shown in Supplementary Table 2, implying that the slab thickness is already converged using even two layers of silver atoms. DDNB alone has been calculated in a large 55×45×35 Ångstrom cell.

The molecule is mainly bound by dispersion on the surface: For PBE+D3, the dispersion part of the interaction yields as much as 297 kJ mol^{-1} (8 × 8, 2 layers), 286 kJ mol^{-1} (10 × 10, 2 layers) or 315 kJ mol^{-1} (8 × 8, 3 layers), while the functional part yields a positive interaction. Nevertheless, the molecule is highly distorted on the surface, and some parts of it are much

closer bound than the others: for example, the oxygen atoms of the nitro group yield distances to their nearest silver atoms of 2.6 Å, whereas the nitrogen of the amine group has a distance of 3.8 Å to its nearest-neighbor silver atom (see Supplementary Figure 4a).

For the climbing-image nudged elastic band[9,10], we used either one, two, or four images to estimate the transition state (Supplementary Figure 4b). To obtain this, we rotated the molecule on an 8x8 cell with a surface thickness of two layers by 60 degrees, optimized both ground states and interpolated the images in between. One image yielded a transition state of 9.3 kJ mol^{-1} (0.096 eV), two images yielded a transition state of 10.0 kJ mol^{-1} (0.104 eV), and four images a transition state of 13.9 kJ mol^{-1} (0.144 eV).

Pivot point determination

In Supplementary Figure 5 the experimental and simulated STM images are compared with the calculated structure of DDNB on Ag(111). The overlaid lattice clearly shows that the rotational centre at the point where an oxygen atom of the molecule is situated directly above an on-top site of the substrate.

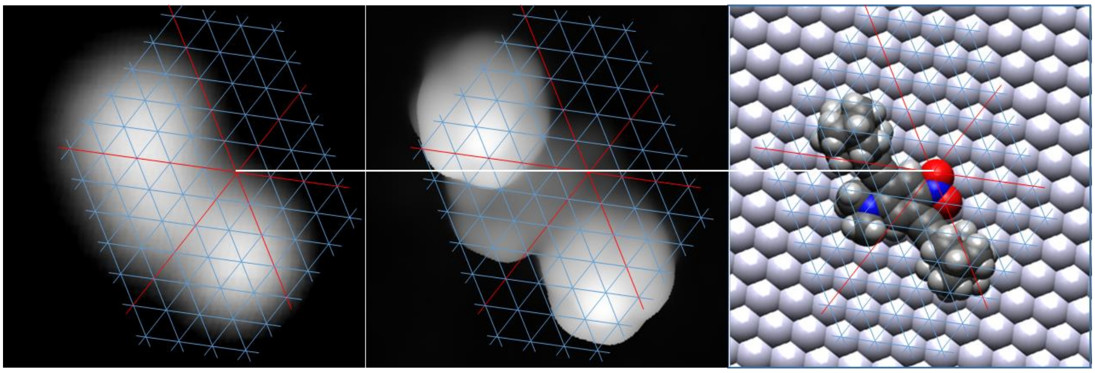

Supplementary Figure 5: Pivot point determination. *Experimental STM image (left), simulated STM image (centre), and calculated structure (right) of DDNB on Ag(111) with lattice grid overlaid.*

Dipole simulation

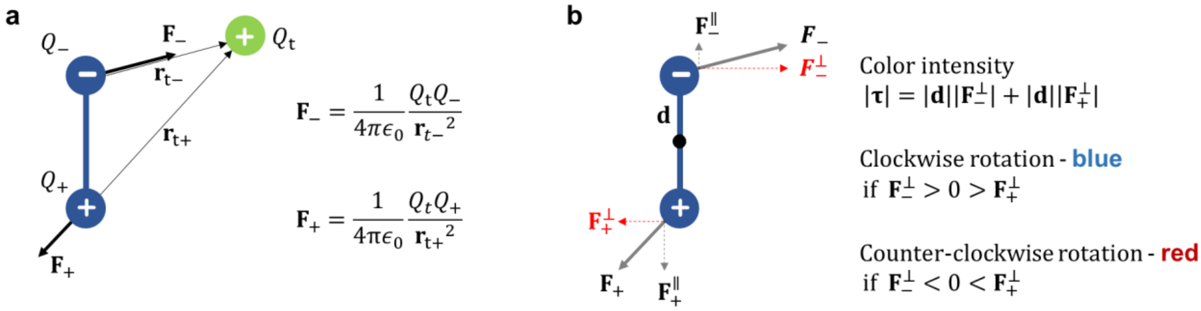

Supplementary Figure 6: Simulation of the torque acting on an electric dipole. *(a) Coulomb forces,* $\mathbf{F}_-$, $\mathbf{F}_+$, *due to a nearby positive test charge on the positive and negative poles of an ideal electric dipole. (b) The assignment of intensity and colour to the simulation shown in Fig. 3a due to the magnitude and direction of the combined torque on the dipole.*

The Coulomb forces (**F**$_-$, **F**$_+$) on each of the charges of the dipole (Q_-, Q_+) due to a positive test charge (Q_t) was calculated for varying positions of the test charge with respect to the dipole (Supplementary Figure 6a). The magnitude of the combined torque, **τ**, on the dipole due to the perpendicular components of **F**$_-$ and **F**$_+$ was then calculated and this value was used to give the pixel intensity in the simulation in Fig. 3a. The colour in Fig. 3a describes the direction of rotation (clockwise/counter-clockwise) and is determined by the direction of the perpendicular forces, as shown in Supplementary Figure. 6b.

Inducing rotation by voltage pulses

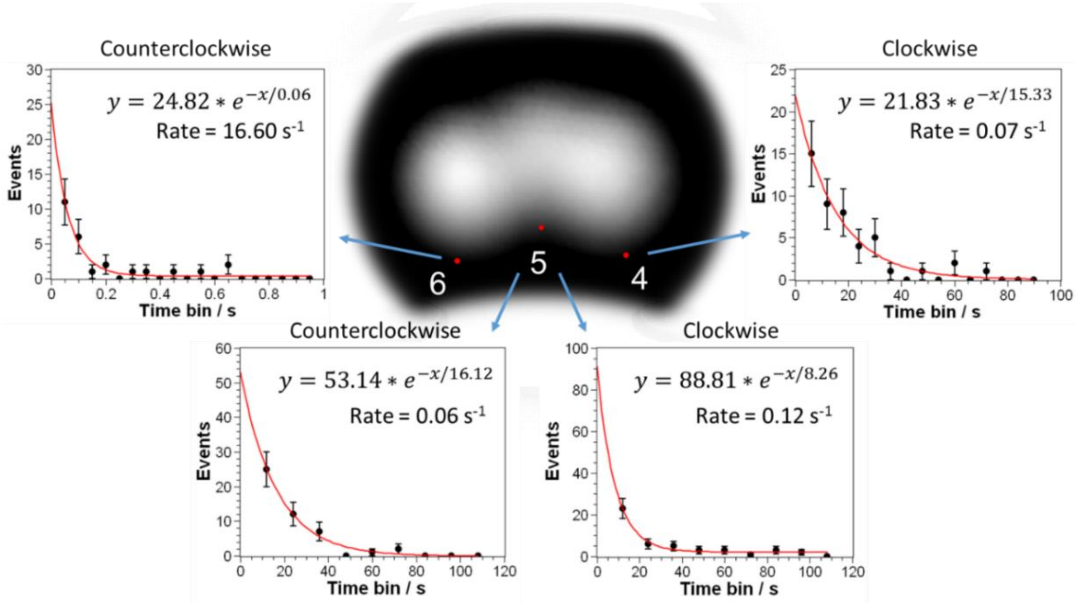

Supplementary Figure 7: Rate of rotation. *Rotation rate at three different positions marked with red dots on the molecule contour. A total of 164 events were recorded, binned, and fitted with exponential decay functions. Error bars represent the standard deviation. Each event corresponds to an open-feedback loop voltage pulse of +1.30 V. Exclusively clockwise/counterclockwise events were observed for positions 4/6. At position 5, 46 clockwise and 47 counterclockwise events were recorded and were treated as separate data-sets.*

The rotation rate of the molecule was determined at the three positions indicated in Supplementary Figure 7. This was done by time-binning a total of 164 rotation events and fitting an exponential decay function. These 164 events included only ±60° rotations; an additional 10 events from the data set involved ±120° events but were not included in determining rates because the directionality of the process cannot be determined from multi-step events. The resulting inverse decay constant (-1/τ) from each fit gives the rate at each position. A voltage of +1.30 V was applied during each pulse. At positions 4 and 6, exclusively clockwise and counterclockwise events, respectively, were observed whereas at position 5 about a 50:50 mixture of both directions resulted. This behavior reflects the conclusions drawn from Fig. 3 in that the position of the tip with respect to the internal dipole of the molecule controls in which direction rotation is induced.

Note that during the voltage pulse from the STM tip the current curve (see Fig. 2b in the main text) typically exhibits one abrupt change only. However, occasionally more than one jump occurs during the voltage pulse, indicating multiple motions, which we excluded from our analysis (in Fig. 3 in the main text) because they cannot be unambiguously assigned to a specific process.

Electrostatic potential

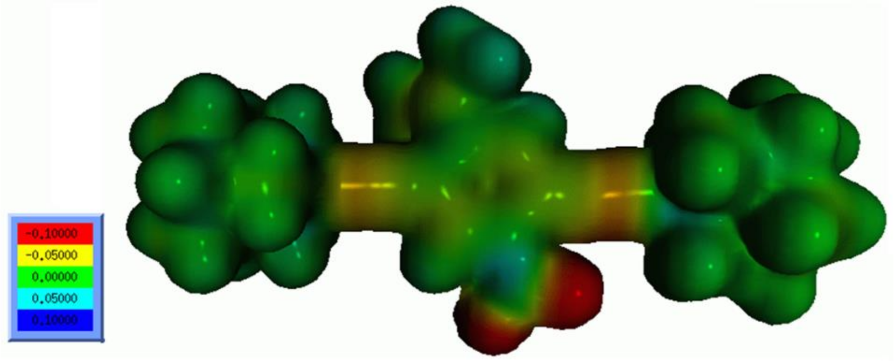

Supplementary Figure 8: Electrostatic Potential (ESP). *ESP charges (colours reflect different potentials, given in units of elementary charge e, as shown in the legend) of the gas phase molecule using isosurfaces of constant electron density, as determined by B3LYP/TZVPPD. The nitro group exhibits a negative charge, whereas the amine group exhibits a small positive charge.*

The calculated electrostatic potential on the molecule (Supplementary Figure 8) shows that the nitro- and dimethylamine- groups hold negative and positive charges, respectively.

Complete rotation sequences in both directions

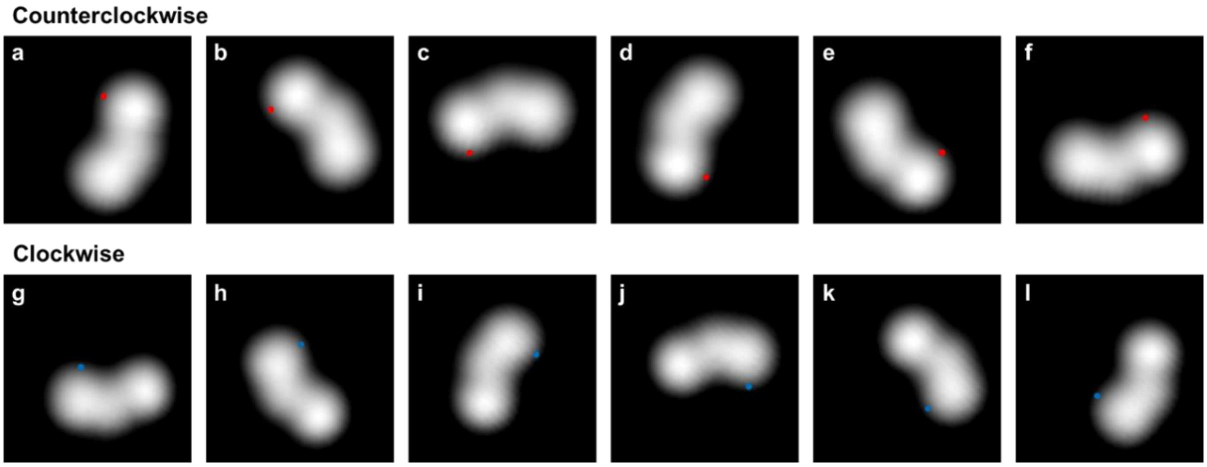

Supplementary Figure 9: Rotation of molecule in clockwise and counterclockwise directions. *Two series of consecutively acquired constant-current STM images following voltage pulses (V = +1.30 V) at the positions marked with dots for counterclockwise (a-f) and clockwise (g-l) rotations of the molecule. All images: I = 0.27 pA; V = 1.03 V; 43 Å × 43 Å.*

The isolated DDNB molecule can be controllably rotated step-wise by 60° in both clockwise and counterclockwise directions. In the examples shown in Supplementary Figure. 9 the tip is positioned at the locations marked in red (counterclockwise) and blue (clockwise) and the bias is increased from the scanning voltage of 1.03 V to 1.30 V. After a short time (typically < 5 s) a single event is recorded in the current signal which corresponds to a 60° rotation upon reimaging. Such a voltage pulse applied in the blue/red locations results exclusively in clockwise/counterclockwise rotation, and hence, the orientation of the molecule can be perfectly controlled.

Rotation sequences with and without Ag Adatom

As seen in Supplementary Figure 10, when the molecule is placed on top of a silver adatom, the pivot point is destabilized and rotation no longer occurs around a single site.

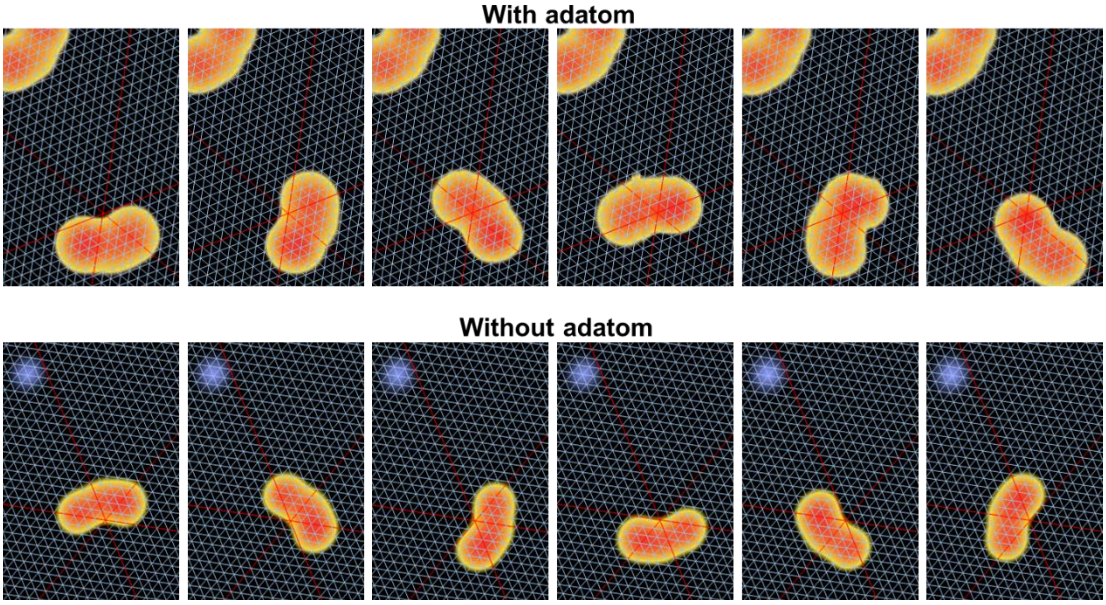

Supplementary Figure 10: Rotation of molecule with and without Ag adatom. *Two series of consecutively acquired constant-current STM images with the initial position of the molecule marked where the red lines in the Ag(111) lattice intersect. The position of the NO_2 group rotates about a single point when no adatom is present, whereas this fixed point of rotation is destabilised when an adatom is present.*

Synthesis of 2,5-di(ethynyladamantanyl)-4-(dimethylamino)nitrobenzene (DDNB)

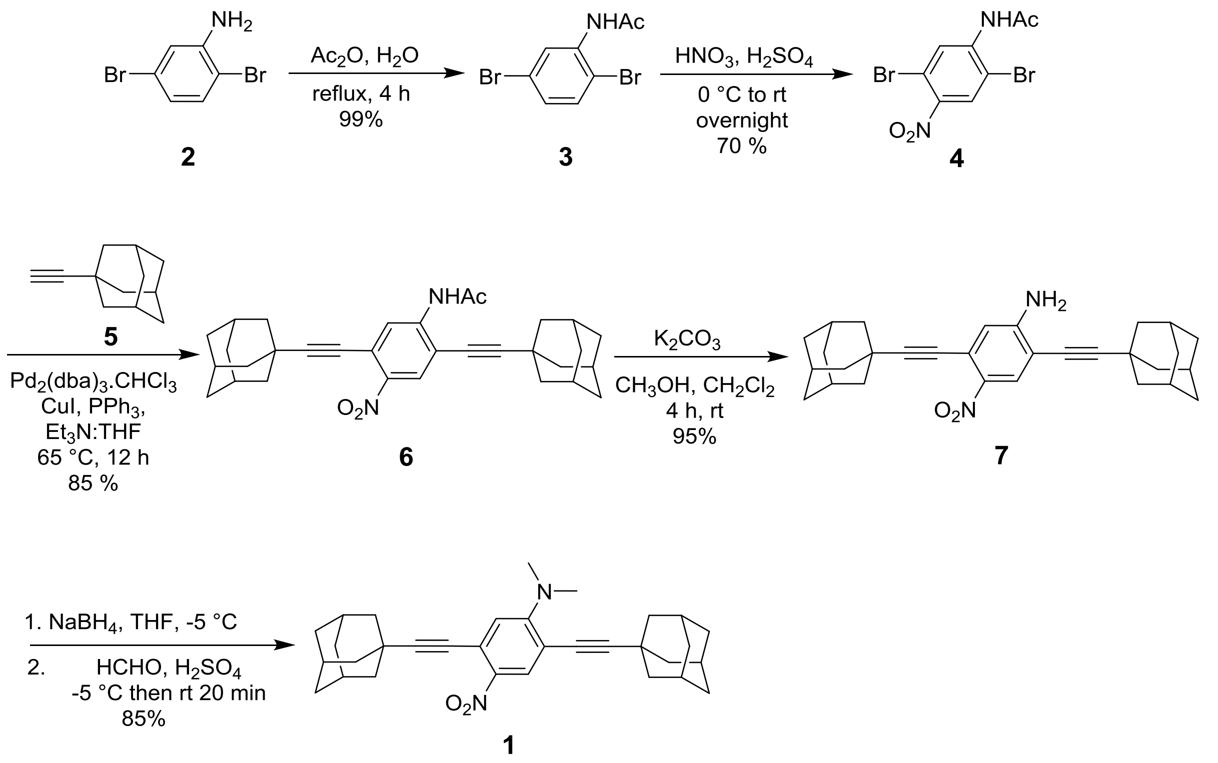

Supplementary Figure 11: Synthesis of DDNB **1**.

General synthetic methods

^{1}H NMR and ^{13}C NMR spectra were recorded at 600 and 150 MHz, respectively. Chemical shifts (δ) are reported in ppm from tetramethylsilane (TMS). All glassware was oven-dried overnight prior to use. Reagent grade tetrahydrofuran (THF) was distilled from sodium benzophenoneketyl under N_2 atmosphere. Triethylamine (Et_3N), and dichloromethane (CH_2Cl_2) were distilled from calcium hydride (CaH_2) under N_2 atmosphere. THF and Et_3N were degassed with a stream of argon for 15 min before being used in the Sonogashira coupling reactions. Palladium-catalyzed reactions were carried out under argon atmosphere. All other chemicals were purchased from commercial suppliers and used without further purification. Flash column chromatography was performed using 230-400 mesh silica gel from EM Science. Thin layer chromatography (TLC) was performed using glass plates pre-coated with silica gel 40 F_{254} 0.25 mm layer thickness purchased from EM Science.

Reported procedures were followed for the synthesis of compounds **3-4**[11] and **5**[12]

DDNB 1. To a solution of **7** (25 mg, 0.055 mmol) in THF (10 mL) was added sodium borohydride powder ($NaBH_4$, 29 mg, 0.77 mmol) and the mixture was cooled to -5 °C. Then a mixture of formaldehyde (CH_2O, 0.5 mL) and sulfuric acid (H_2SO_4, 2.0 mL) was added dropwise maintaining the same temperature. After the addition was complete, the mixture was stirred at room temperature for 20 min. The reaction was quenched with 15 mL of an

ethanol/water (1:1 v/v) mixture and the organic phase was extracted with diethyl ether (Et_2O, 3 X 20 mL). The organic extract was dried over anhydrous magnesium sulfate ($MgSO_4$) and the solvent was removed in vacuo. The residue was purified by column chromatography (silica gel, 3% EtOAc in hexanes) to provide **1** as pale yellow solid (22 mg, 85%). ^{1}H NMR (600 MHz, $CDCl_3$) δ 8.08 (s, 1H), 6.75 (s, 1H), 3.10 (s, 6H), 2.00 (br s, 12H), 1.96-1.94 (m, 6H), 1.75-1.70 (m, 12 H). ^{13}C NMR (150 MHz, $CDCl_3$) δ 156.26, 140.42. 132.45, 120.47, 120.07, 112.08, 106.56, 105.59, 77.79, 76.70, 42.69, 42.63, 42.47, 36.50, 36.48, 30.82, 30.66, 28.06, 28.05. HRMS (ESI) *m/z* calcd. for $[M+H]^+$ $C_{32}H_{38}N_2O_2$ 483.3006, found 483.2998

Compound 6. An oven dried 10 mL screw-cap tube equipped with a stir bar was charged under argon flow with compound **4** (300 mg, 0.89 mmol), adamantane **5** (356 mg, 2.22 mmol), $Pd_2(dba)_3{\cdot}CHCl_3$ (92 mg, 0.089 mmol), PPh_3 (117 mg, 0.45 mmol), and CuI (34 mg, 0.178 mmol). Freshly distilled and degassed THF (3.75 mL) and NEt_3 (1.25mL) were added, the screw-cap tube was sealed, and the reaction mixture was stirred at 70 °C for 12 h. After cooling to rt, the reaction was quenched with saturated $NH_4Cl_{(aq)}$ (20 mL) and extracted with CH_2Cl_2 (2 X 30 mL). The organic phase was washed with water (30 mL), dried over anhydrous $MgSO_4$, filtered, and the filtrate was concentrated under vacuum. The crude product was purified by column chromatography (silica gel, 5-10% EtOAc in hexanes) to provide **6** as a yellow solid (331 mg, 85%)**.** ^{1}H NMR (600 MHz, $CDCl_3$) δ 8.60 (s, 1H), 8.04 (br s, 1H), 8.03 (s, 1H), 2.25 (s, 3H), 2.06-2.03 (m, 3H), 2.0-1.97 (m, 15 H), 1.77-1.74 (m, 6H), 1.73-1.71 (m, 6H). ^{13}C NMR (150 MHz, $CDCl_3$) δ 168.27, 144.50, 141.91, 127.79, 123.26, 120.50, 111.73, 109.13, 108.46, 75.91, 73.14, 42.80, 42.30, 36.45, 36.26, 30.84, 30.79, 28.00, 27.90, 25.12. HRMS (ESI) *m/z* calcd. for $[M+H]^+$ $C_{32}H_{36}N_2O_3$ calc. 497.2799, found 497.2790

Compound 7. In a 50 mL round-bottom flask precursor **6** (200 mg, 0.40 mmol) was dissolved in CH_2Cl_2 (10 mL). Methanol (10 mL) and K_2CO_3 (553 mg, 4.0 mmol) were added and the mixture was stirred at room temperature for 4 hours. The solvents were evaporated under vacuum and the product was purified by column chromatography (silica gel, 3% EtOAc in hexanes) to provide **7** as a yellow solid (173 mg, 95%).^{1}H NMR (600 MHz $CDCl_3$) δ 8.06 (s, 1H), 6.73 (s, 1H), 4.66 (br s, 2H), 2.01-1.95 (m, 18H), 1.73-1.71 (m, 12H). ^{13}C NMR (150 MHz, $CDCl_3$) δ 150.90, 140.03, 129.77, 120.91, 117.88, 107.77, 106.72, 106.69, 76.41, 73.81, 42.98, 42.46, 36.48, 36.39, 30.79, 30.67, 28.05, 28.03 HRMS (ESI) *m/z* calcd. for $[M+H]^+$ $C_{30}H_{34}N_2O_2$ $[M+H]^+$ 455.2693, found 455.2687.

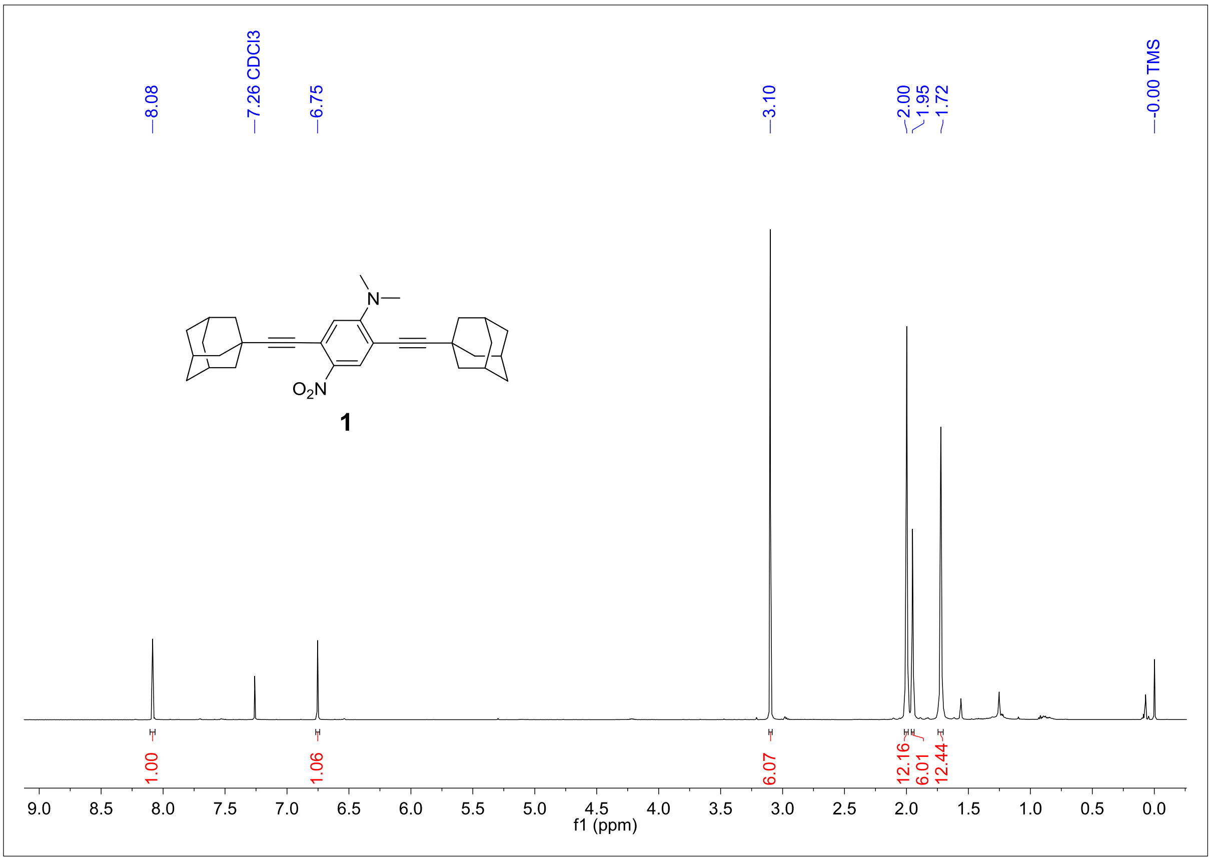

Supplementary Figure 12: ^{1}H NMR of **1**

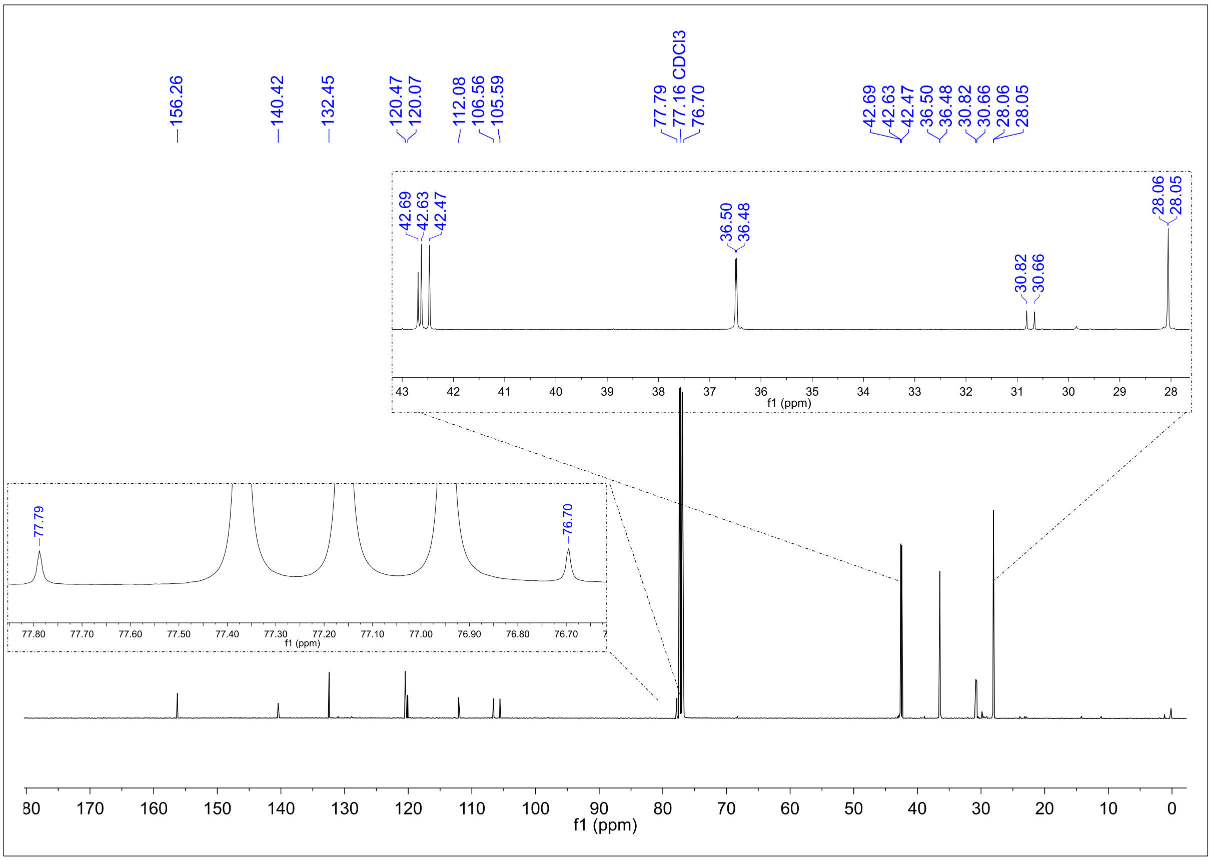

Supplementary Figure 13: ^{13}C NMR of **1**

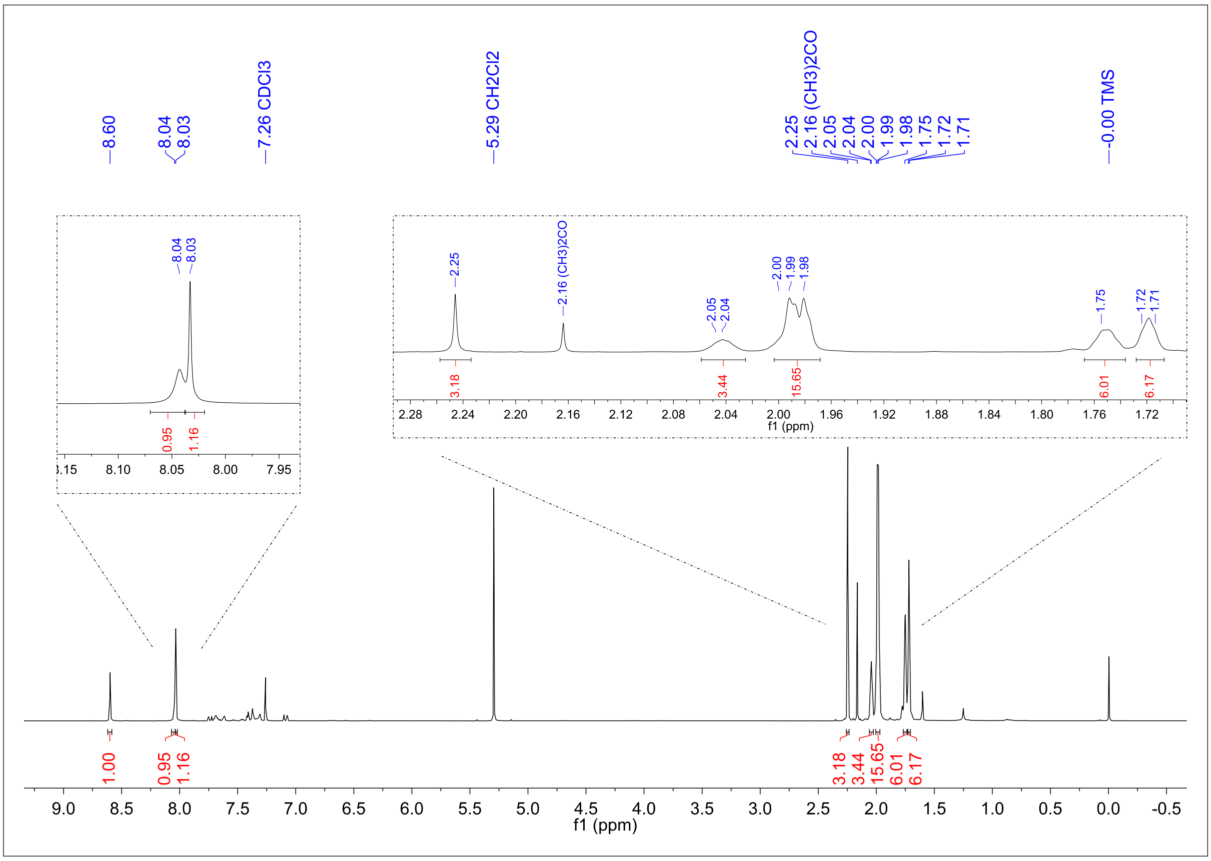

Supplementary Figure 14: ^{1}H NMR of **6**

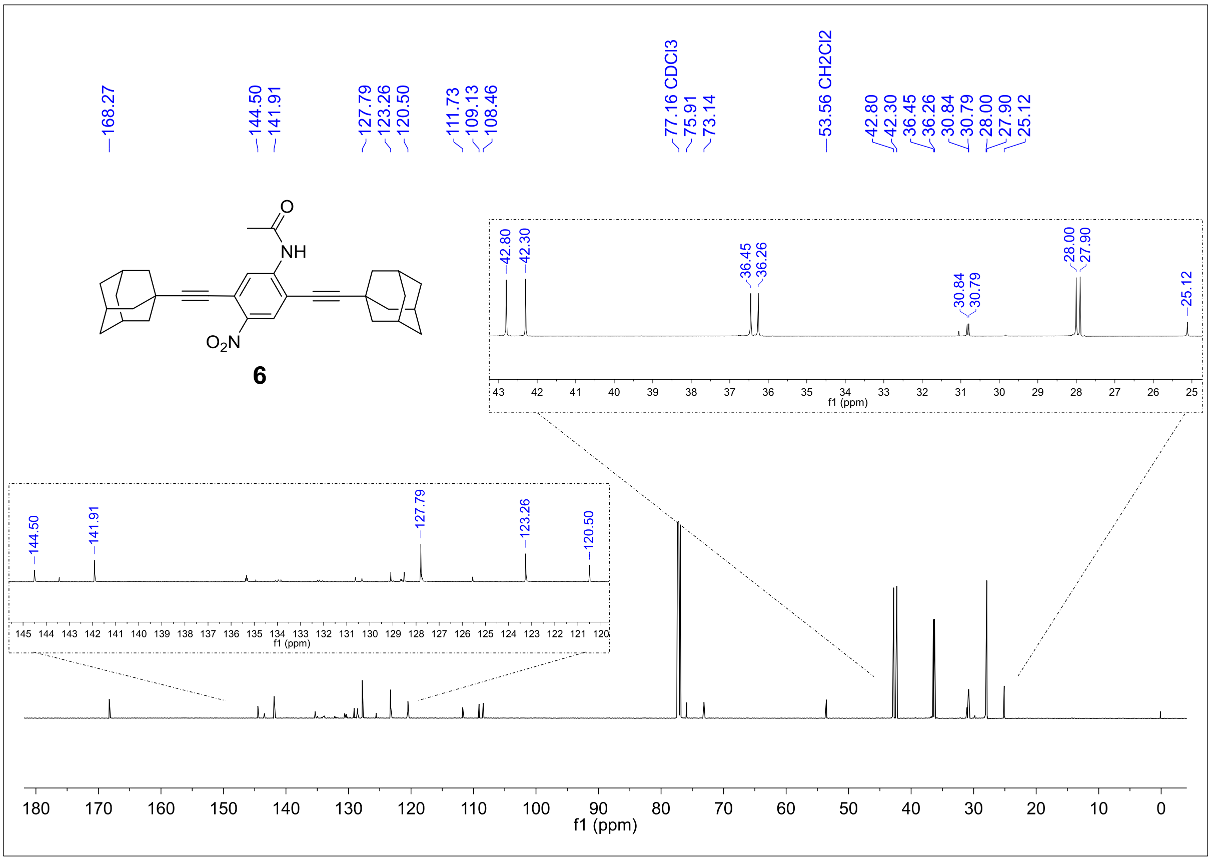

Supplementary Figure 15: ^{13}C NMR of **6**

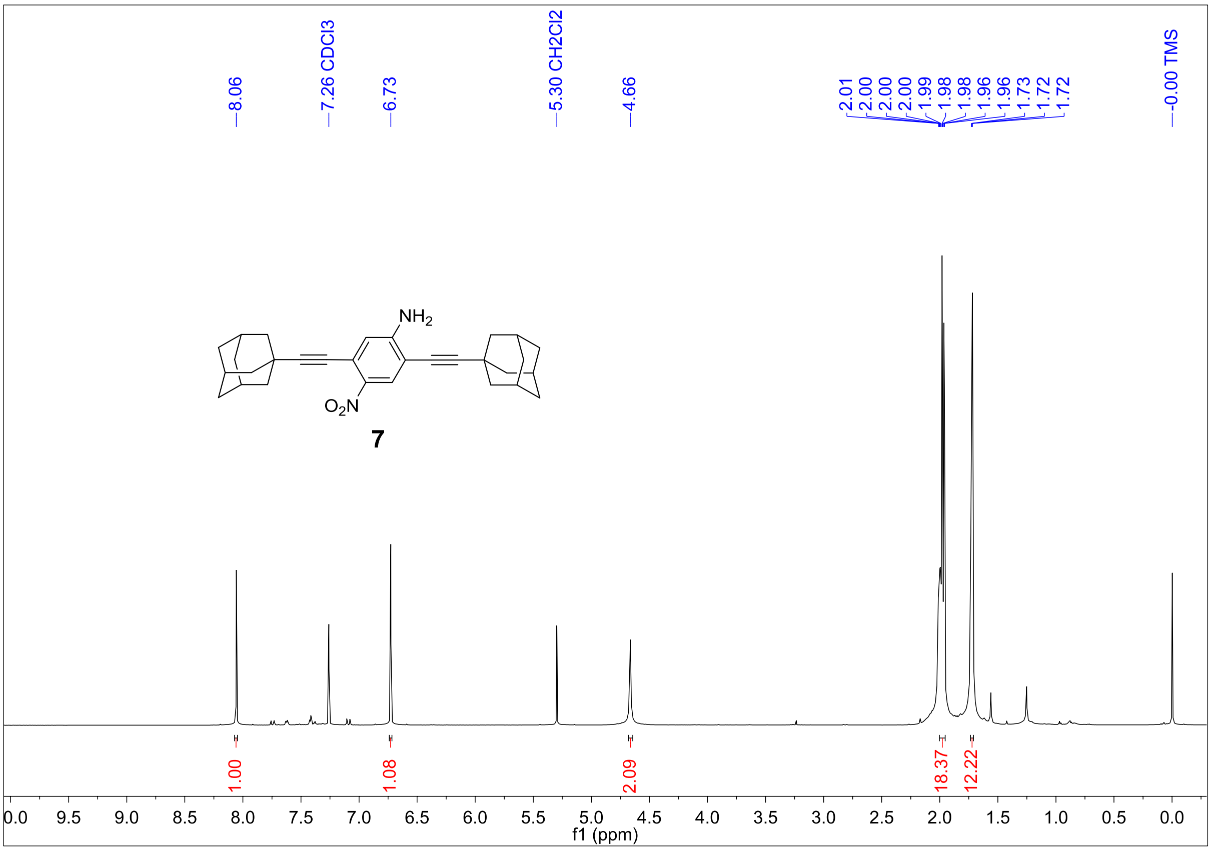

Supplementary Figure 16: ^{1}H NMR of **7**

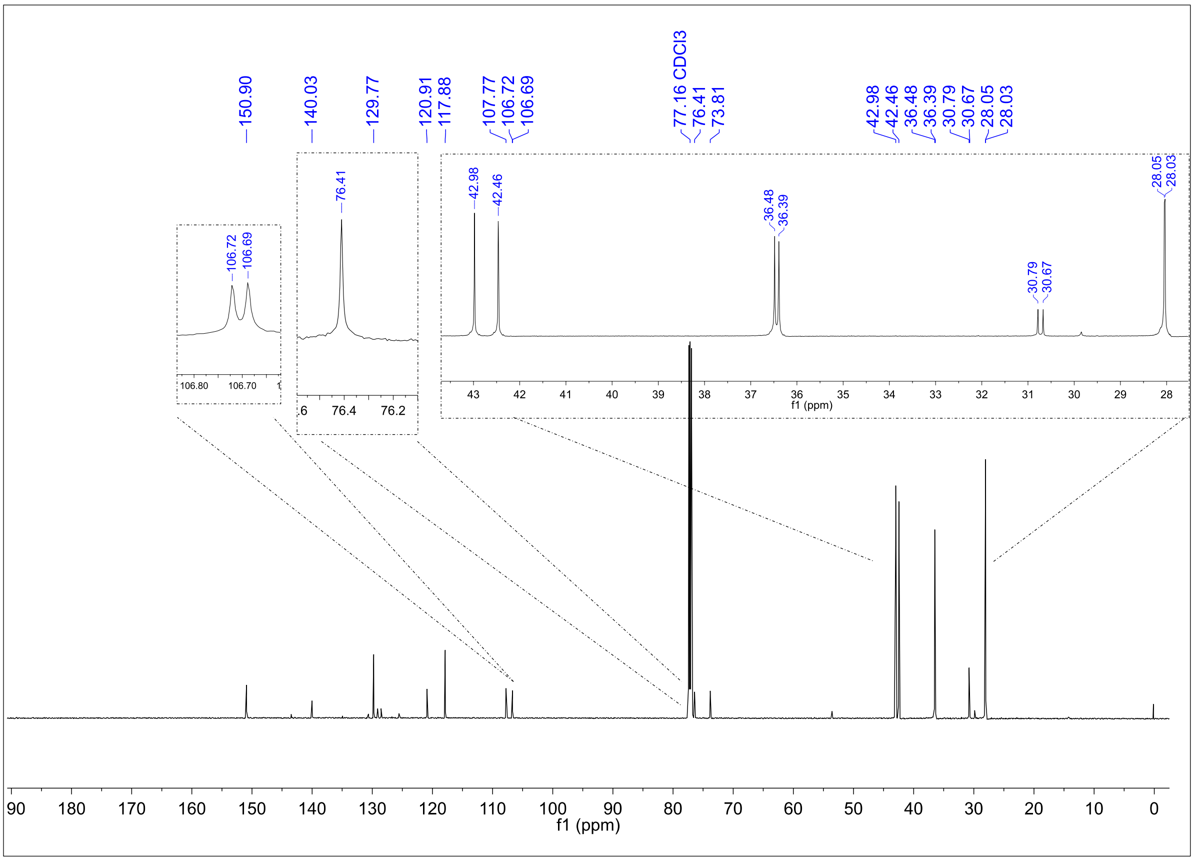

Supplementary Figure 17: ^{13}C NMR of **7**

Supplementary References

[1] Kresse, G. *et al*. VASP 5.4.1, http://www.vasp.at, accessed 3.5.2018.

[2] Kresse, G. & Joubert, D. From ultrasoft pseudopotentials to the projector augmented-wave method. *Phys. Rev.* **59**, 1758 (1999).

[3] Perdew, J. P., Burke, K. & Ernzerhof, M. Generalized gradient approximation made simple. *Phys. Rev. Lett.* **77**, 3865 (1996).

[4] Grimme, S., Antony, J. & Ehrlich, S. A consistent and accurate ab initio parametrization of density functional dispersion correction (DFT-D) for the 94 elements H-Pu. Krieg, H; *J. Chem. Phys.* **132**, 154104 (2010).

[5] TURBOMOLE version 6.5, COSMOlogic; 2011 developed by R. Ahlrichs et al., see www.turbomole.de, accessed 3.5.2018

[6] Rappoport, D. & Furche, F. Property-optimized Gaussian basis sets for molecular response calculations. *J. Chem. Phys.* **133**, 134105 (2010).

[7] Stephens, P. J., Devlin, F. J., Chabalowski, C. F. & Frisch, M. J. Ab initio calculation of vibrational absorption and circular dichroism spectra using density functional force fields. *J. Phys.Chem.* **98**, 11623 (1994).

[8] Becke, A. D. Density-functional thermochemistry. III. The role of exact exchange. *J. Chem. Phys.* **98**, 5648 (1993).

[9] Henkelman, G., Uberuaga, B. P. & Jónsson, H. A climbing image nudged elastic band method for finding saddle points and minimum energy paths. *J. Chem. Phys.* **113**, 9901 (2000).

[10] Henkelman, G. & Jónsson, H. Improved tangent estimate in the nudged elastic band method for finding minimum energy paths and saddle points. *J. Chem. Phys.* **113**, 9978 (2000).

[11] Tour, J. M., Rawlett, A. M., Kozaki, M., Yao, Y., Jagessar, R. C., Dirk, S. M., Price, D. W., Reed, M. A., Zhou, C.-W., Chen, J., Wang, W. & Campbell, I. Synthesis and preliminary testing of molecular wires and devices. *Chem. Eur. J.* **7**, 5118 (2001).

[12] Chu, P.-L., Wang, L.-Y., Khatua, S., Kolomeisky, A. B., Link, S. & Tour, J. M. Synthesis and single-molecule imaging of highly mobile adamantane-wheeled nanocars. *ACS Nano* **7**, 35 (2013).
